# Smartphone-based imaging systems for medical applications: a critical review

**DOI:** 10.1117/1.JBO.26.4.040902

**Published:** 2021-04-15

**Authors:** Brady Hunt, Alberto J. Ruiz, Brian W. Pogue

**Affiliations:** Dartmouth College, Thayer School of Engineering, Hanover, New Hampshire, United States

**Keywords:** smartphone, smartphone imaging, smartphone systems, point-of-care, handheld, mobile

## Abstract

**Significance:** Smartphones come with an enormous array of functionality and are being more widely utilized with specialized attachments in a range of healthcare applications. A review of key developments and uses, with an assessment of strengths/limitations in various clinical workflows, was completed.

**Aim:** Our review studies how smartphone-based imaging (SBI) systems are designed and tested for specialized applications in medicine and healthcare. An evaluation of current research studies is used to provide guidelines for improving the impact of these research advances.

**Approach:** First, the established and emerging smartphone capabilities that can be leveraged for biomedical imaging are detailed. Then, methods and materials for fabrication of optical, mechanical, and electrical interface components are summarized. Recent systems were categorized into four groups based on their intended application and clinical workflow: *ex vivo* diagnostic, *in vivo* diagnostic, monitoring, and treatment guidance. Lastly, strengths and limitations of current SBI systems within these various applications are discussed.

**Results:** The native smartphone capabilities for biomedical imaging applications include cameras, touchscreens, networking, computation, 3D sensing, audio, and motion, in addition to commercial wearable peripheral devices. Through user-centered design of custom hardware and software interfaces, these capabilities have the potential to enable portable, easy-to-use, point-of-care biomedical imaging systems. However, due to barriers in programming of custom software and on-board image analysis pipelines, many research prototypes fail to achieve a prospective clinical evaluation as intended. Effective clinical use cases appear to be those in which handheld, noninvasive image guidance is needed and accommodated by the clinical workflow. Handheld systems for *in vivo*, multispectral, and quantitative fluorescence imaging are a promising development for diagnostic and treatment guidance applications.

**Conclusions:** A holistic assessment of SBI systems must include interpretation of their value for intended clinical settings and how their implementations enable better workflow. A set of six guidelines are proposed to evaluate appropriateness of smartphone utilization in terms of clinical context, completeness, compactness, connectivity, cost, and claims. Ongoing work should prioritize realistic clinical assessments with quantitative and qualitative comparison to non-smartphone systems to clearly demonstrate the value of smartphone-based systems. Improved hardware design to accommodate the rapidly changing smartphone ecosystem, creation of open-source image acquisition and analysis pipelines, and adoption of robust calibration techniques to address phone-to-phone variability are three high priority areas to move SBI research forward.

## 1 Introduction

**Fig. 1:**
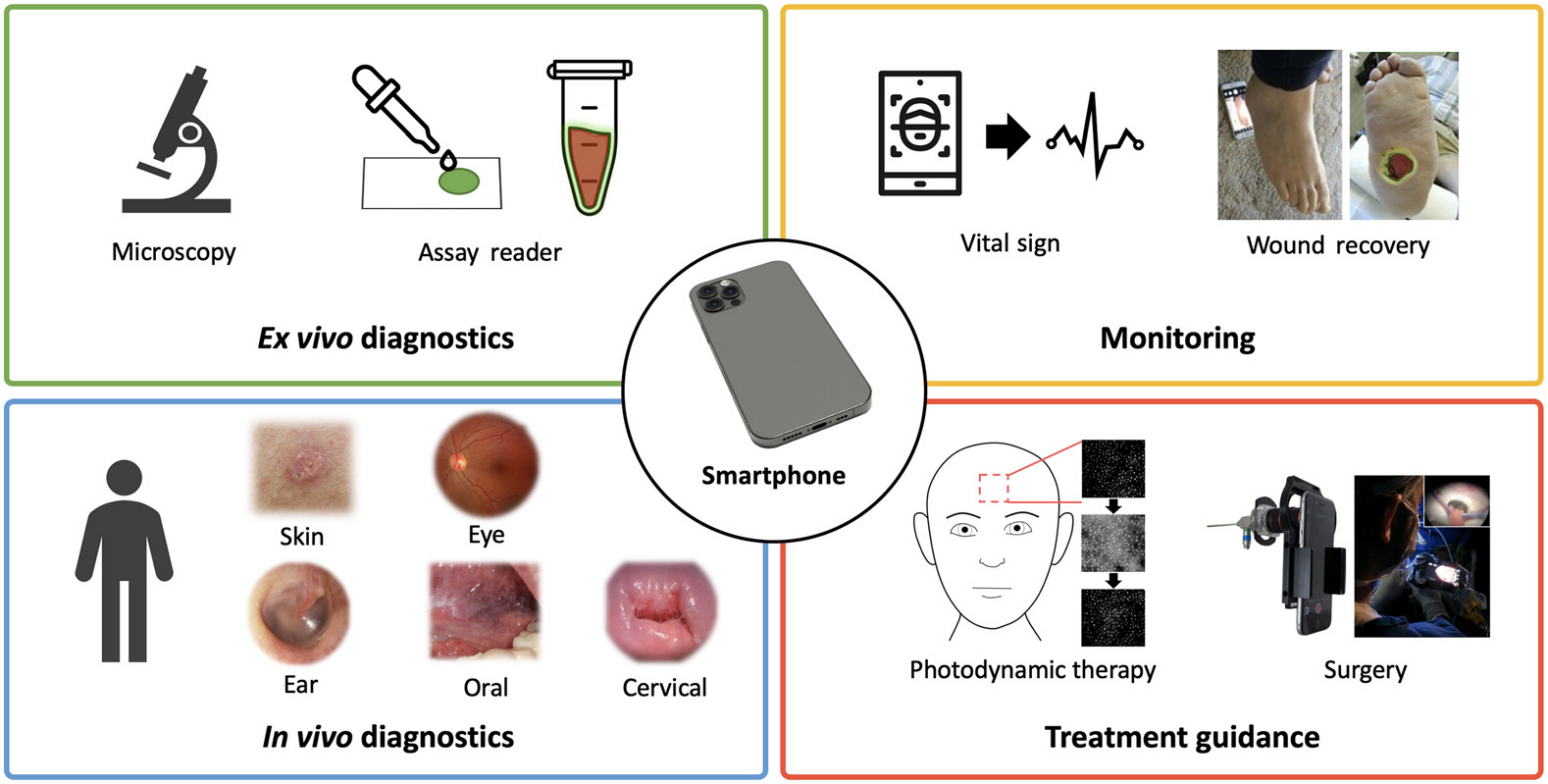
SBI for various biomedical imaging applications grouped into four clinical workflows.

Smartphone-based imaging (SBI) has been proposed for numerous biomedical applications, many of which use an optical attachment to augment or extend the native device capabilities (Fig. 1). In the past decade, the most common application for SBI has been diagnostic analysis of *ex vivo* specimens (i.e., point-of-care testing), which has utilized smartphones in a variety of microscopy and microfluidic detection schemes.^1^^–^^4^ SBI is also frequently proposed for noninvasive monitoring and diagnosis of externally accessible tissues, particularly in dermatological applications.^5^ More recently, SBI for minimally invasive procedures and treatment guidance has also been reported, including photodynamic therapy (PDT),^6^^–^^9^ endoscopy,^10^^–^^13^ *in vivo* microscopy,^14^^–^^17^ and surgery.^18^^–^^22^ As *ex vivo* diagnostic applications have been extensively reviewed elsewhere,^1^^–^^4^ this review focuses on SBI systems for real-time tissue imaging applications (i.e., *in vivo* monitoring, diagnosis, and treatment guidance). However, recent developments in *ex vivo* diagnostic system designs, which may have relevance in tissue imaging applications are also discussed for comparison and contrast.

The review is structured as follows. First, the established and emerging smartphone capabilities as well as methods and materials for SBI system interface design are reviewed, with an eye toward classifying them as to their optical, mechanical, and electrical components. Each of these can be passive in their functionality to simply extend what the phone camera itself is doing, or they can be active, in terms of adding function that the SBI itself could not achieve. Then, the emerging applications of SBI systems are presented within the three aforementioned roles of tissue imaging (monitoring, diagnosis, and treatment guidance). Finally, the pros and cons of smartphone utilization in emerging applications are discussed alongside recommendations to improve clinical translation and uptake of research advances in SBI.

## 2 System Interface Design

SBI systems leverage built-in sensors of modern smartphones in addition to various optical, mechanical, electrical, and software components to augment native device capabilities. When developing smartphone-based optical instrumentation, a fundamental design choice is how custom hardware and software will interface with the smartphone. Current SBI system interfaces vary greatly at both the hardware and software levels, ranging from basic utilization of the unmodified smartphone camera with built-in or third party software to application-specific optical attachments being actively controlled with custom software. The terms “smartphone-based” and “using a smartphone” appear frequently in biomedical optics research abstracts but do not adequately capture the diversity of the underlying interface designs and degree of smartphone utilization. In this section, the first discussion is on the built-in capabilities of modern smartphones that can be leveraged for biomedical imaging applications. Then, characterization of systems from the literature is done in terms of the hardware and software componentry utilized to augment built-in capabilities, highlighting commonly used materials and methods for developing SBI systems.

### 2.1 Built-in Capabilities

**Fig. 2:**
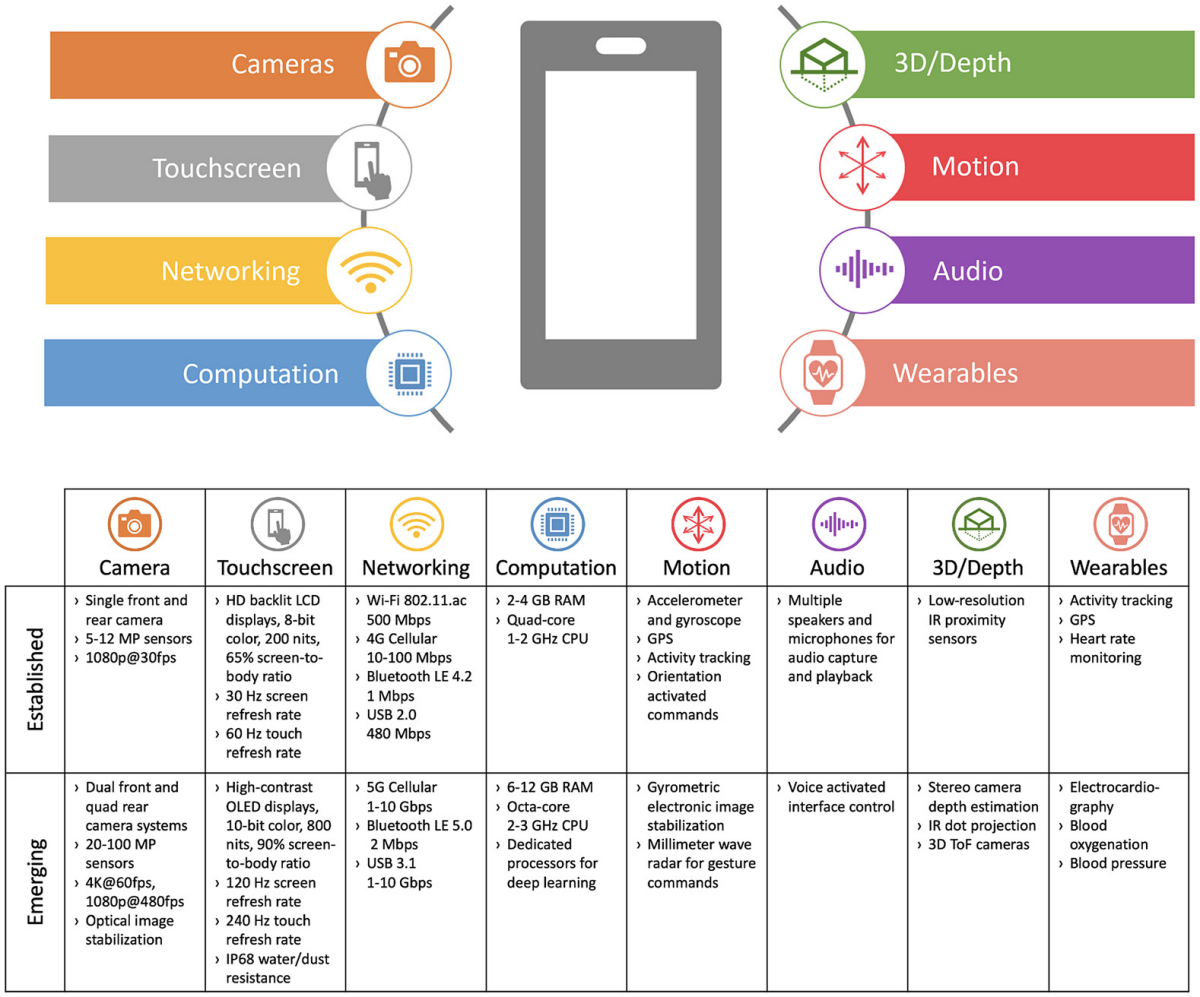
Established and emerging smartphone capabilities for biomedical imaging applications.

Driven by global demand for mobile computing and telecommunications, smartphones have been at the forefront of consumer electronics innovation for nearly two decades. As a result, built-in sensor capabilities continue to evolve at a rapid pace, making smartphones the “Swiss Army knife” of mobile computing. Using an internet database,^23^ we compared smartphone specifications for several iOS and Android smartphones released over the past decade and created a summary of eight established and emerging capabilities which are relevant for biomedical imaging applications (Fig. 2). We defined established capabilities as specifications which have been available for over 5 years and are common for current entry-level smartphones, whereas emerging capabilities are those available only on current high-end smartphones and may become more widely available in the future.

#### 2.1.1 Cameras

Camera technology continues to be an area of fierce competition and innovation among smartphone manufacturers. Modern smartphones are equipped with several compact camera modules for front and rear photoacquisition at various magnifications. These preassembled modules are small form factor optical systems typically consisting of a multielement lenses, apertures, optical filters, CMOS sensors, and motors for autofocus and image stabilization.

The primary engineering constraint is the small form factor of the sensor and lens elements. Most smartphones utilize ∼1/3” format sensors (active pixel area $∼17\;{\mathrm{mm}}^{2}$) with between 5 and 12 MP (∼1.1 to 1.8  μm pixels). Newer models are moving toward larger ($∼{1/1.3}^{\prime \prime }$, $∼65\;{\mathrm{mm}}^{2}$ active pixel area) and ultrahigh-resolution sensors (50+ MP with ∼2.4  μm effective pixels after processing). These high-resolution CMOS sensors now support “4K,” or 2160 p, video acquisition at up to 60 fps, as well as ultrafast acquisitions at lower resolution (1080 p at 240 fps and 720 p at 960 fps).

Another major trend for smartphone cameras in the past few years has been a shift from a single to multiple rear cameras with additional lenses for ultrawide, macro, and telephotoacquisition. Periscope lenses are becoming more common and use folding mirror geometries to achieve longer focal lengths and as high as 10× optical zoom. Equivalent focal lengths listed by manufacturers currently range from around 18 mm for ultrawide lenses all the way up to 240 mm for the longest periscope lenses, with most primary widefield lenses being in the 25 to 30 mm range. Adjusting for a ∼3- to 10-fold crop factor based on the ${1/4}^{\prime \prime }$ to ${1/1.3}^{\prime \prime }$ sensor format range, actual focal lengths for current smartphones range from around 3 to 30 mm. Having a variety of lenses with the possibility for multicamera acquisition has not been extensively utilized in SBI systems but could prove useful for biomedical imaging applications.

In conjunction with larger sensors and multilens systems, there has been movement toward more sophisticated integrated signal processing for image denoising and reconstruction using multiple acquisitions. While these advances in computational photography may be advantageous for some applications, the lack of fine-grained control of image acquisition and processing pipelines is not ideal for medical and scientific imaging. Over the last few years smartphones have gained the ability to fix imaging parameters and access the unprocessed (RAW) imaging data, which is essential for quantitative imaging applications. Section 2.3 provides a detailed discussion on these advances of imaging acquisition controls.

#### 2.1.2 Other optical sensors

Current smartphones are also equipped with other built-in and/or peripheral optical technologies which can be utilized for biomedical imaging applications including: ambient light, proximity/depth, thermal, and wearable sensors. Recent advances and references to additional topical reviews of these sensors are summarized in this section.

In contrast with cameras, ambient light sensors are simple photodetectors which only measure the intensity of incident light. The primary purpose of ambient light sensing (ALS) is to automatically adjust the user screen brightness based on lighting conditions; however, several reports have demonstrated use of this sensor to measure intensity changes due to light attenuation^24^^,^^25^ as well as light emission^26^ from chemical assays. A recent review on the use of ALS in point-of-care testing stated that it can provide “resolution as low as 0.01 lux over a wide range of wavelengths from 350 to 1050 nm.”^27^

Low-resolution proximity sensors to detect when a phone is being held close to the face/ear have been on smartphones for over a decade but have had little utility for biomedical applications. However, on-board depth sensing technologies and associated software development kits to support augmented reality are becoming more capable. Depth estimation using dual-camera stereoscopic images has been utilized to segment background from foreground objects and to create depth of field maps for synthetic “bokeh.” Although stereo depth estimations have good spatial resolution, they are relatively low-precision and susceptible to variability in lighting/imaging conditions.^28^ Light-field imaging is an emerging alternative to stereoscopic imaging but has been evaluated in a relatively small number of studies and has not yet to be integrated in commercial systems.^29^^–^^31^ Most recently, 3D time-of-flight cameras are an emerging capability which can potentially provide depth information at sufficiently quantitative spatial and temporal resolutions for dynamically measuring distances and volumes.^32^^,^^33^ Ulrich et al.^34^ provided a comprehensive review of these emerging methods and other RGB-D sensing technologies.

In addition to depth sensing, compact IR cameras have also enabled thermal imaging on smartphones. Two commercially available attachments are the FLIR One Pro and SEEK CompactPRO. Beyond clip-on thermal attachments, some phone models, such as the Caterpillar CAT S61, have now incorporated on-board thermal imaging sensors.^35^ Kirimtat et al.^36^ recently conducted a head-to-head comparison of the FLIR and SEEK attachments and broadly summarized related works in biomedicine, which use both smartphone-based and nonsmartphone-based handheld thermography. This review includes applications using the FLIR One smartphone sensor in both diagnostic and treatment guidance applications.^22^^,^^37^^–^^41^

Wearable optical sensors that wirelessly interface with smartphones hold great potential for more consistent and noninvasive health monitoring.^42^ Established capabilities for wrist-based sensors are activity and heart rate monitoring, which use motion and optical sensing, respectively. In recent years, newer devices have added sensing capability for electrocardiography, skin temperature, blood oxygenation, and blood pressure monitoring. Wearable systems have been extensively reviewed elsewhere.^43^^–^^47^ This review focuses on approaches which utilize native smartphone optical sensors or otherwise use the smartphone to actively control an external optical system, as opposed to approaches that passively acquire point-based optical measurements.

#### 2.1.3 Ancillary capabilities (touchscreen, networking, motion, and computation)

Other built-in capabilities that distinguish smartphones from traditional computing platforms include touchscreen displays, networking, motion/audio interface control, and computational power. Here, we briefly summarize advances in these areas which may play a role in SBI systems moving forward.

Modern smartphones provide high-performance displays which can be versatile interfaces for many applications. Displays with 10-bit color could provide advantages compared to established 8-bit displays for applications involving high dynamic range imaging. Newer displays can also achieve higher display and touch refresh rates (up to 120 and 240 Hz, respectively), which could be helpful in applications where image data need to be played back and/or annotated with high temporal fidelity.

Wireless networking is another great strength of smartphones, which can facilitate untethered handheld imaging. Mature communications protocols are well supported on smartphones including Wi-Fi 802.11.ac, 4G cellular networks, and Bluetooth low-energy. In applications where device-to-device communication is needed, Bluetooth can support relatively low-latency communication (<100  ms) at up to 2 Mbps and over large distances (100 to 400 m).^48^ In cases where even lower latency and higher bandwidth device-to-device communication is needed, wired USB connections can be utilized for up to 10 Gbps and submillisecond latency.

Motion and audio sensors are well established on smartphones but have not yet been widely utilized for biomedical imaging applications. Motion sensors will continue to play a role in photography image stabilization as well as for 3D depth sensing. One area for consideration in medical imaging is the use of motion and audio for contactless user interfaces through gesture or voice commands. For example, millimeter wave radar is an emerging sensing technology which could enable enhanced, 360-deg hand gesture recognition for user interface control.^49^^,^^50^

Embedded computing architectures continue to improve and enable more data intensive applications on smartphones, including image processing pipelines. However, improvements in mobile computing performance do not readily translate for biomedical imaging applications without appropriate software frameworks to support full utilization of the hardware. This remains a barrier in the research community as computationally efficient image analysis on smartphones requires significant programming expertise. However, machine learning-based image analysis could provide a relatively versatile, easy-to-use, and computationally efficient strategy compared to traditional image processing toolkits, which are not well supported on mobile devices. Dedicated processors to support accelerated machine learning inference are likely to become a standard moving forward.

### 2.2 Hardware

**Fig. 3:**
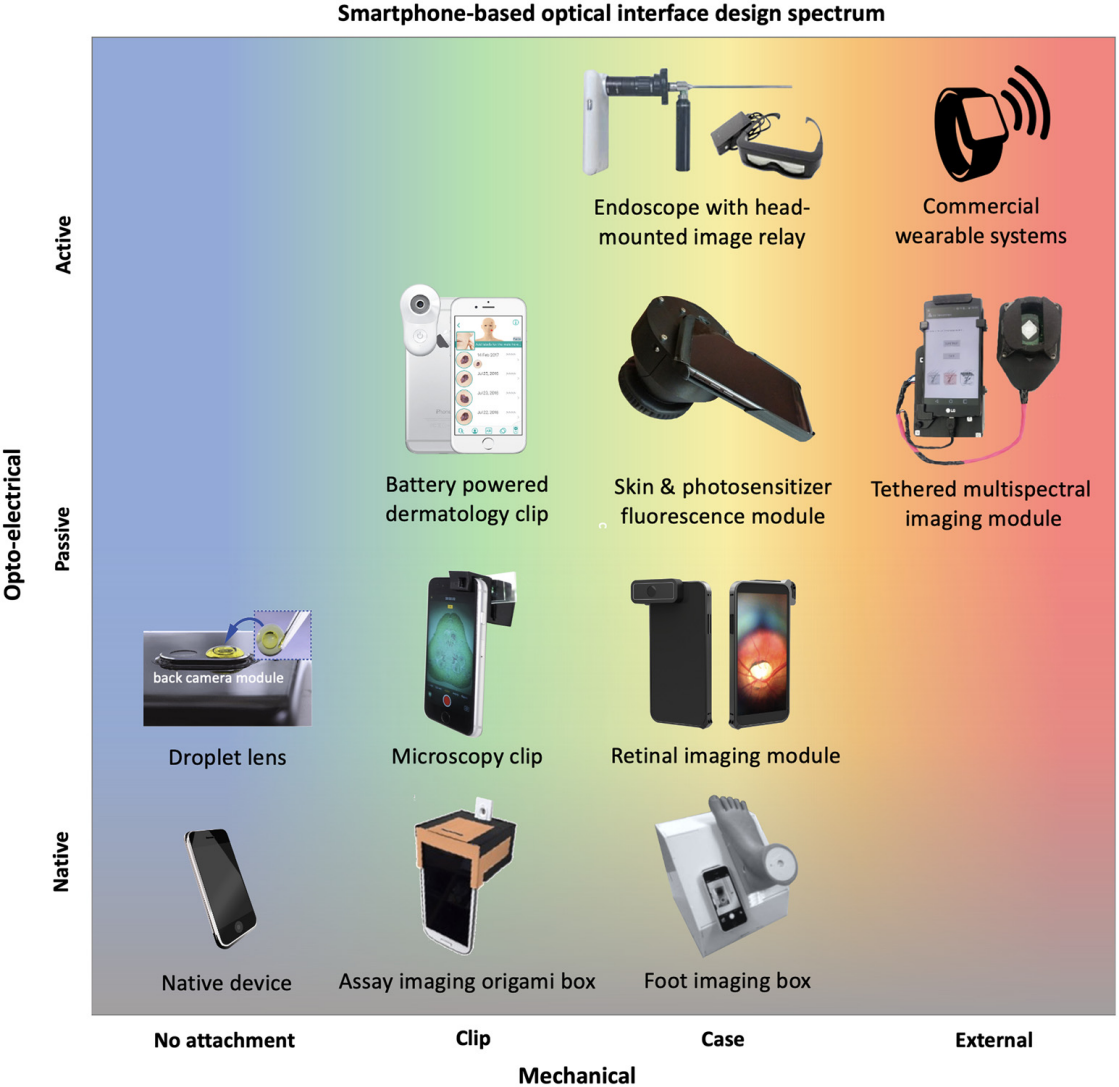
Smartphone-based optical interface design spectrum. The spectrum of optical interfaces for smartphone-based biomedical imaging spans from use of the native device only to fully external optical systems that interface with the phone via wired/wireless communication. Attachment designs vary along mechanical and optoelectronical axes from minimal/passive attachments to more complex and actively controlled attachments. Examples of commercial and research prototype systems are plotted within the spectrum for reference. Visuals adapted from the following sources: assay imaging box,^54^ foot imaging box,^55^ droplet lens,^56^ microscopy clip,^57^ retinal imaging module,^58^^,^^59^ dermatology clip,^60^ photosensitizer fluorescence module,^6^ endoscope,^10^ and multispectral imaging module.^52^

Hardware design of SBI systems ranges along a design spectrum from passive interfaces with minimal adaptation of built-in optics to active interfaces with battery-powered, smartphone-controlled optical attachments. Figure 3 illustrates this spectrum with examples from research literature as well as commercial products. SBI systems occupy the space between a native smartphone camera system and a fully external optical sensing system, such as a wrist-based wearable device. Moving up and to the right, more sophisticated hardware interfaces which can enable a greater system control and optical performance are observed; however, these designs often become phone-specific and potentially diminish the longer term stability of optical designs in integrating with newer smartphones. Advances in 3D printing have been instrumental in overcoming this challenge for research prototyping, but further developments to address the scalability of SBI hardware interfaces is important to achieve greater impact and translation. For example, some designs minimize the reliance on smartphone hardware by interfacing external optical systems through wireless or wired connections to the phone.^13^^,^^51^^,^^52^ Recently, Alawsi and Al‐Bawi reviewed smartphone-based adapter designs across a large variety of both *ex vivo* and *in vivo* point-of-care imaging applications.^53^ Here, we characterize common materials and methods utilized to create optical, mechanical, and electrical interfaces for SBI systems with particular attention to considerations for the *in vivo* applications covered in this review.

#### 2.2.1 Optical

The primary optical interface for SBI systems is naturally the built-in camera. Smartphone cameras have been modified using off-the-shelf optical components including lenses (spherical, aspheric, achromats, infinity corrected objectives, Fresnel lenses, and reversed smartphone lenses from disassembled camera modules), filters (bandpass, longpass, neutral density, polarizers), apertures, mirrors (folding, scanning, and dichroic), optical fibers for light relay of flash LED, diffusers, and diffraction gratings. Integration of these passive optics in front of the smartphone camera is a fairly straightforward; however, it does impose some design limitations. One major challenge toward computer-aided design of SBI systems is variability and/or unspecified optical properties of built-in lenses, filters, and LEDs of preassembled smartphone camera modules. Bae et al.^10^ approximated the smartphone lens of their system for ray tracing simulation using the crop factor of the CMOS sensor, the fixed aperture specified by the vendor, and assuming the lens is set to infinite-focus. In both *in vivo* and *ex vivo* microscopy applications, others have utilized an reversed smartphone lens to match the light collection angle of the built-in lens phone and relay distortion-free conjugate images to the CMOS sensor that fill the entire sensor.^16^^,^^57^^,^^61^^,^^62^ For reproducible calibration of spectral response across different smartphone models, several reports have utilized commercially available reference color targets (X-rite ColorChecker for example) to apply phone-by-phone calibration factors.^63^^–^^69^ Quantitative methods for calibration of SBI systems have also been proposed.^70^

One area that is not as well appreciated is control of stray light in SBI systems. Systems for low light level applications require exclusion of ambient room lighting to preserve the signal specificity or purity. This is especially important in spectroscopic, chromatic, and filtered light applications for external tissue measurements.^6^^,^^17^^,^^52^^,^^64^^,^^68^^,^^71^^–^^73^ Additionally, light emission from tissue comes at high numerical aperture, and so control over access to these signals requires careful lens design and external light control. Filtering of signals is always challenging given the range of choices and the typically short camera–tissue distances, and so evaluation of the contaminating signals is important, as are choices about use of potentially multiple filters.^6^

Future developments in fabrication of customized optics could enable greater flexibility in smartphone optical interface design.^74^^,^^75^ Miniaturized polymer “droplet” lenses for both light collection^76^^,^^77^ and filtering^56^^,^^78^ are a promising development as they can potentially be fabricated at very low cost and assembled with minimal adaptation of smartphone optics. Rapid advances in computational imaging and optical design also hold great promise to be utilized in conjunction with existing smartphone optical sensors or to be added through additional dedicated sensors.^79^^–^^84^

#### 2.2.2 Mechanical

Mechanical interfaces for SBI systems serve several functions depending on the use case including optical alignment and coupling of custom optics to the smartphone, background light rejection in fluorescence applications, and ergonomic setup and clinical use. Custom enclosures for optical attachments are typically fabricated using 3D printing to accommodate unique smartphone geometries. Fused filament printing is most common and typically provides alignment precision within a few hundred microns. Stereolithography 3D printing is beginning to be more widely used and can fabricate enclosures with sub-100  μm precision. This is often adequate for positioning most optical components but may not be ideal for lens alignment, particularly high numerical aperture lenses. For applications with custom lenses needing more precise alignment, cage rod assemblies mounted inside a 3D-printed enclosure have been used.^15^^,^^16^

In terms of attachment mechanisms, SBI systems can be broadly categorized into one of three categories: (1) no attachment, (2) clip attachment, and (3) case attachment. SBI systems with no attachment utilize only the native device or otherwise may communicate wirelessly with external optics. For example, He and Wang demonstrated Weiner estimation to reconstruct “pseudohyperspectral” images in an attachment-free manner,^68^ whereas Cai et al.^51^ demonstrated a smartphone-interfaced wireless spectrometer for *in vivo* measurement of biosamples. Both approaches can, in principle, work for various smartphone models without requirement for hardware customization.

Clip attachments designs intended be lower profile, less dependent on a specific phone geometry, and easily attached/detached from the smartphone. Clip attachments for the top of the phone near the cameras and the base of the phone using the charging port have both been demonstrated.^57^^,^^85^^,^^86^ Spigulis et al.^87^ demonstrated a sticky platform-like attachment that was not as low profile as other clip designs but enabled easy attachment/detachment of various smartphones. Such designs are appealing in the sense that they could work with multiple phones; however, clip attachments that include custom lens assemblies are often impractical as manual alignment of lenses with the phone camera is cumbersome and error prone.

A more common design for SBI systems with custom optics is a case attachment. Case attachments accommodate larger optics/electronics systems and more precisely couple the system to the camera. As smartphone manufacturers often published detailed specifications for third party manufacturing of phone cases, 3D design of case attachments is straightforward. Their primary drawback is that they often require design modifications for each smartphone model, and with the rapid succession of models produced today, there would be frequent changes needed for matching the updates. This change in phone shape with successive models on an annual basis is one of the most difficult challenges in these attachment device approaches.

Sterilizability or sanitation is another important and potentially overlooked mechanical design constraint. Attachments that are easily assembled/disassembled are preferable for frequent sterilization or cleaning. For applications where SBI systems are used by contact, use of biocompatible materials should be encouraged. Most 3D-printed prototypes do not comply with these needs and so merely serve as a rapid prototype that needs to be implemented in a medical grade material production. In sterile clinical environments, such as the surgical suite, clear sterile plastic sleeves are commonly used for camera and microscope systems.^18^^,^^20^

#### 2.2.3 Electrical

Some SBI systems also use embedded electronics to facilitate controlled light delivery, active optical components (scanning mirrors, tunable filters, and motorized mounts), wireless/wired data relay, and for microcontroller logic. Such active attachment designs are more common for fluorescence and multispectral imaging applications for more controlled light delivery.^52^^,^^66^^,^^88^^–^^90^ For example, Cavalcanti et al.^88^ used a multiplexed system of fiber optics to deliver colored light from eight different LED sources across the visible spectrum to the tip of an otoscope. Wired connections have also been used to interface smartphones with USB cameras for tethered capsule endoscopy as well as multispectral dermal imaging.^13^^,^^52^ Cai et al.^51^ developed a pencil-like spectrometer based on a compact WiFi-enabled camera. Currently, the use of wired/wireless communication to embedded electronics seems somewhat underutilized and is one promising avenue to increase control and customization of SBI optical systems.

### 2.3 Software

**Fig. 4:**
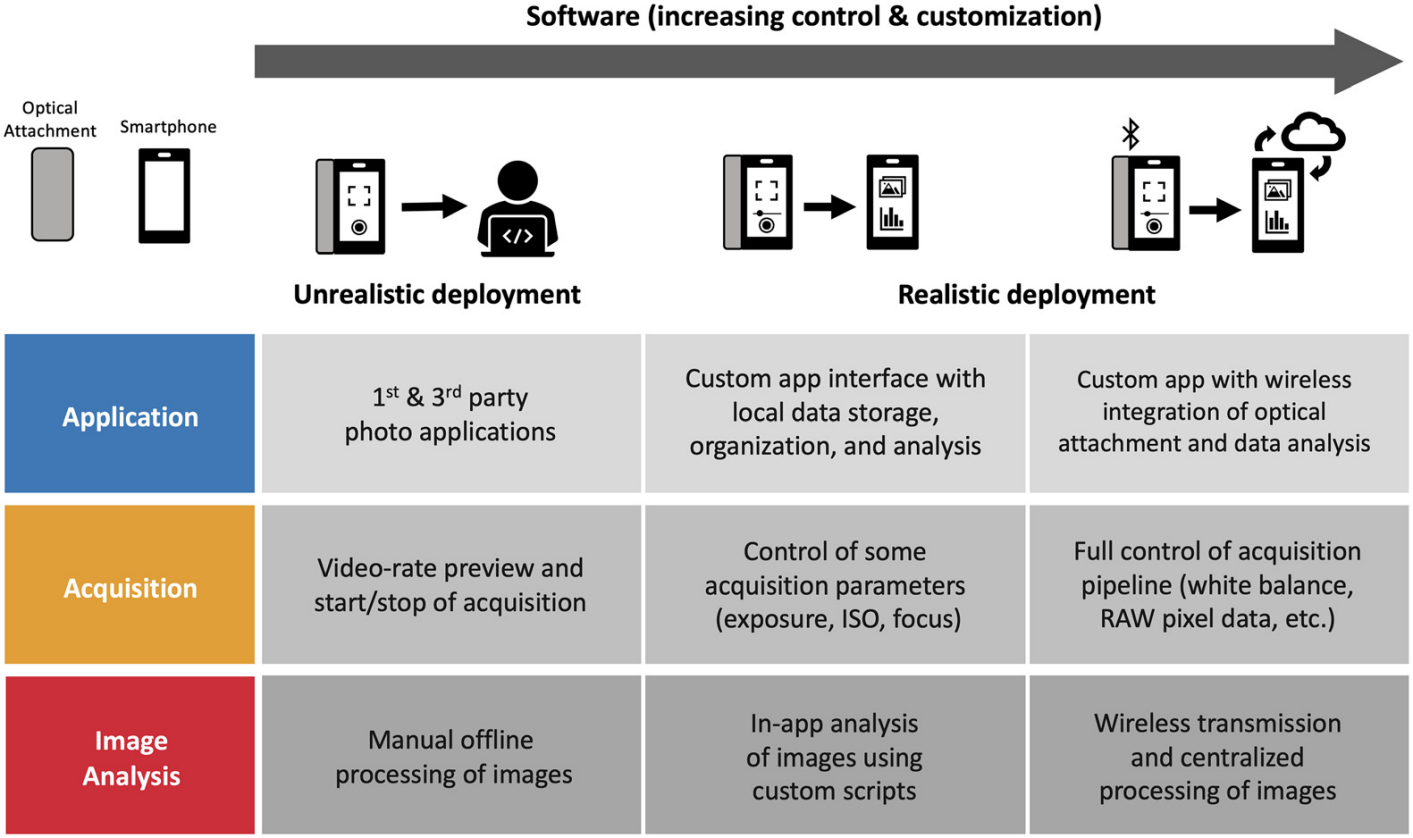
Smartphone-based software design spectrum. Approaches and core functionalities supported by SBI systems in unrealistic and realistic deployments with increasing degrees of customization are highlighted. Unfortunately, the vast majority of SBI systems reported in the current literature use unrealistic acquisition and analysis pipelines.

In terms of software interfaces, there is a great deal of variety in the level of functionality supported in research prototypes, with many systems relying on third party camera software and manual postprocessing of images. Figure 4 highlights approaches and core functionalities of software supported by SBI systems in unrealistic and realistic deployments with increasing degrees of control and customization. Clinical deployment of SBI systems for real-time use into clinical settings necessitates custom software to facilitate both data acquisition and analysis. State-of-the-art SBI software interfaces should accomplish both of these functions in addition to facilitating easy operation for the end user.

Acquisition is one area where smartphones have great potential to streamline and enhance the usability for biomedical imaging. The core functionalities of good acquisition software are fivefold: (1) provide a video-rate preview of the camera sensor data, (2) control relevant acquisition parameters (focus, exposure, gain, white balance, RAW pixel data, etc.), (3) trigger start and stop of camera acquisition, (4) facilitate storage and organization of acquired data (by patient or sample, for example), and (5) interface with downstream image analysis pipelines (either through on-board or wireless communication). Despite smartphones having perfected these functionalities for everyday photography, these advances may not readily transfer to SBI systems with customized optics and/or acquisition pipelines.

As medical providers are often multitasking while performing clinical examinations, real-time visualization, and easy triggering of acquisition is of particular importance for diagnostic and treatment guidance systems. For example, Bae et al.^10^ developed a custom app for their endoscopy system, which facilitates image relay to a head-mounted display. Other SBI systems that facilitate improved ease of use and contactless acquisition through hand-gesture or voice-commands.^91^^–^^93^ The highly networked capabilities of smartphones seem largely underutilized, given the potential ability to interface with many peripherals at the same time, via multiple communication protocols (i.e., Bluetooth, Wi-Fi, cellular, or direct cable connection).

Another crucial function for acquisition software in scientific and medical applications is the capability to control acquisition and postprocessing parameters, retain adequate bit depth, and interface with downstream image analysis pipelines. The ability to acquire reproducible images has been an ongoing challenge for smartphone-based systems due to their “point-and-shoot” design which automates imaging acquisition and postprocessing.^94^ Autofocusing/exposure is generally beneficial for improved usability of imaging systems; however, developers should be careful of relying on high-level native and third party libraries to provide this functionality as it may inhibit quantitative reproducibility. In recent years, the ability to fix imaging parameters and access the unprocessed RAW pixel data has become more accessible on both iOS and Android platforms. This has been leveraged in more recently reported systems for improved quantitative accuracy in low-light applications, although incorporating RAW image processing capability in downstream on-board analysis on phones has not yet been demonstrated.^6^^,^^16^^,^^64^^,^^73^

In terms of analysis, common functions for SBI systems include image review/consultation,^91^^,^^95^ region of interest selection,^19^^,^^96^ colorimetric quantification,^73^^,^^97^ intensity measurements,^6^^,^^98^^,^^99^ diagnostic classification,^65^^,^^71^^,^^90^^,^^100^ and segmentation of tissue structures.^14^^,^^15^^,^^55^^,^^101^ However, implementing custom image analysis routines on smartphone operating systems requires substantial programming expertise, leading researchers to continue to rely on manual image processing workflows. The problem is further exacerbated by phone-to-phone variability, requiring additional work to ensure smartphone-based analysis routines function properly for multiple phone cameras and operating systems. Both one-time calibration of phone cameras using optical targets as well as per-measurement calibration by measuring ambient lighting conditions have been proposed to improve quantitative reproducibility.^68^^,^^73^^,^^86^^,^^94^^,^^102^ An alternative solution to phone-by-phone calibration for image analysis pipelines could be to leverage cloud-based computing with deep learning. Such an approach has been demonstrated by Song et al.^100^ for oral imaging. Centralizing image data acquired from multiple users/phones to an appropriate privacy-compliant server enables collection of larger, more diverse datasets which can in turn be used to develop more robust image analysis pipelines and deploy them without having to continually update smartphone software.

## 3 Context and Applications

As noted in Sec. 1, SBI systems have been proposed for a variety of diagnostic applications and are increasingly being proposed for noninvasive monitoring of disease conditions. There are also emerging reports proposing the use of SBI to guide treatment procedures (surgical or PDT) or to conduct more invasive diagnostic imaging of deep tissues (endoscopy). This section contains a structured summary of recent reports using SBI tissue imaging for three types of applications: monitoring, diagnosis, and treatment guidance. While there are some commonalities in how SBI could be advantageous in all three of these categories, understanding of the clinical context (i.e., where is the measurement being taking and who is taking it) is important in assessing the strengths and weaknesses of smartphone utilization. As there are some SBI systems and applications, which may well fit into more than one of the three aforementioned categories, note that this categorization was not intended to be a rigid one, but rather a broad grouping for purposes of discussion.

### 3.1 Monitoring

Monitoring applications often require repeated sampling over a sustained period (from hours up to months) to assess appreciable differences in disease states, and therefore benefit from being low-cost and noninvasive. In recent years, SBI systems have been proposed for monitoring of vital signs,^103^^–^^109^ blood glucose,^110^^–^^113^ blood pressure,^114^^,^^115^ blood oxygenation,^68^^,^^116^ hemoglobin concentration,^72^ atrial fibrilation,^117^^,^^118^ jaundice,^73^^,^^97^^,^^119^^,^^120^ skin cancer,^121^ and diabetic foot ulcers.^55^^,^^122^ All of these applications propose utilizing either contact-based or contactless optical measurements using the smartphone camera, most often by individuals on themselves (i.e., self-monitoring). With the exception of blood glucose monitoring, all are proposed for use through installation of a software app onto native devices and do not require external optical attachments.

Vital sign monitoring using contact-based video recording of fingers (i.e., photoplethysmography) was an early SBI application proposed for smartphones,^103^ and there are continued reports of novel ways to utilize this approach to extract additional hemodynamic metrics (blood pressure, oxygenation, cardiac arrhythmia, etc.).^115^^,^^116^^,^^118^ However, this is clearly one area where practical considerations of the context have been overlooked and smartphone utilization is increasingly questionable, as to the use case. Although contact-based vital sign monitoring is achievable using smartphone cameras, continuous monitoring is infeasible without dedicating the smartphone for that purpose. Low-cost, dedicated wearable sensors which can relay data to the smartphone are clearly a more practical and reliable long-term solution for obtaining contact-based vital sign measurements.

Noncontact-based methods for extracting hemodynamics using video recording are a more recent development which more fully utilizes the spatial information provided by smartphone imaging.^68^^,^^72^^,^^104^^,^^123^^,^^124^ Two recent works, by Park et al.^72^ and He and Wang,^68^ used computational techniques to infer higher resolution spectral responses and predict hemoglobin content in tissue using only RGB smartphone image data. Park et al. evaluated their method on a dataset acquired from 153 participants referred for a blood count at an academic health center in Kenya, whereas He and Wang performed their assessments on a few volunteers in a “dark” lab environment. Park et al. also performed a more rigorous quantitative comparison of their noninvasive hemoglobin concentration prediction compared to the gold-standard obtained by venous blood draw, observing good quantitative agreement of their noninvasive hemoglobin predictions ($R^{2}=0.91$ for 15 test patients).^72^ The work by Park et al. showed a rigorous and realistic assessment of their method in a clinical setting. However, both methods required careful calibration of smartphone camera spectral response, used post-hoc image analysis routines, and were only conducted by research personnel, so it remains to be seen if they can be effectively deployed at scale on smartphones.

Other monitoring application for SBI involves photographic surveillance of dermal conditions, skin cancer, or diabetic foot ulcers for example. In these applications, there is less need for optical instrumentation, but rather software design to facilitate standardized acquisition, automated analysis, and data relay to clinical providers as needed. For example, in the case of diabetic foot ulcers, Yap et al.^125^ developed an app that provides a “ghost outline” of the user’s foot based on prior measurements to ensure that repeated measurements were well coregistered. And Ploderer et al.^93^ proposed the use of the front facing camera and voice guidance to enable users to acquire photos of the bottom of their feet more conveniently. While less novel in their extraction of optical tissue properties, these approaches demonstrate a focus on real-world usability in SBI monitoring applications.

### 3.2 Diagnosis

Diagnostic measurements of tissue are typically performed to examine a suspicious area of tissue more closely to assess the underlying cause of abnormality and/or severity of disease. As diagnostics are typically used for triage to treatment interventions, such applications more likely to be performed by medical personnel and in clinical settings rather than by individuals at home. Some of the primary *in vivo* diagnostic modalities proposed for SBI systems include white light imaging,^52^^,^^65^^,^^66^^,^^91^^,^^100^^–^^102^^,^^126^^–^^133^ autofluorescence imaging,^17^^,^^65^^,^^66^^,^^71^^,^^100^ multispectral/hyperspectral imaging,^52^^,^^64^^,^^88^ endoscopy,^10^^–^^13^ *in vivo* spectroscopy,^14^^,^^51^^,^^85^ and *in vivo* microscopy.^14^^–^^16^^,^^62^^,^^134^ For these modalities, the most frequent imaging sites are external tissues (dermis, facial, and retinal), externally accessible tissues (oral cavity, cervix, and ear), as well as some deeper tissues in the case of endoscopy (bladder, larynx, and esophagus).

In past years, a number of novel SBI systems have been developed for the application of skin cancer diagnosis and surveillance through smartphone-based dermascopy. In 2016, Kim et al. published one of the first smartphone-based multispectral imaging systems and explored its potential use for dermal lesion assessment.^89^ Their system consisted of a motor-controlled wheel of optical filters placed in front of the LED flash with embedded electronics and custom app to synchronize acquisition of a spectral image cube from images acquired at nine different wavelengths from 440 to 690 nm. They validated their spectral measurements against a non-SBI liquid-tunable-crystal-filter system using a colored optical target and performed exploratory normal volunteer imaging at imaging two dermal sites (one acne and nevus region). Acquisition speed was not reported, but image cube processing speed was reportedly 30 s on the phone and could be sped up to 3 s per image cube with cloud-based processing. More recently, Uthoff et al. reported a multispectral system that performs sequential illumination using eight different colored LEDs across the visible to near-infrared regime (450 to 940 nm) and was actively controlled using a custom smartphone app.^52^ Their system implemented two different camera acquisition methods: one using the built-in camera and one using a tethered USB camera. The authors performed measurements using both camera setups and semiquantitatively assessed skin chromophores (hemoglobin, oxygenated hemoglobin, and melanin) in two clinical cases (one benign and one malignant). Acquisition reportedly required 20 s per image cube, and it was not specified whether on-board image cube analysis was achieved. While both systems are state-of-the-art implementations in terms of integration of custom hardware and software into novel, compact imaging systems, their primary shortcoming is a lack of integration into a practical clinical workflow to more concretely demonstrate the advantages of having these modalities on an SBI system. This can be done but requires more extensive testing than was reported.

Endoscopic procedures using both rigid and flexible optical probes have incorporated into SBI systems for otolaryngological, esophageal, and cervical examination of epithelial tissues.^10^^–^^15^^,^^135^ In the context of endoscopy applications, handheld systems with rigid optical probes seem more synergistic with smartphone utilization as the probe and the device are compact enough to simultaneously manipulate and position.^10^^,^^135^ By contrast, other microendoscope systems utilize thin flexible coherent fiber bundles for image relay.^14^^,^^15^ These systems benefit less from smartphone utilization, as the optical probe is typically manipulated independently of the monitor screen and the optical probes can relay images over longer distances (often several feet), reducing the need for an ultracompact optical enclosure. Similarly, tethered capsule endoscopes benefit minimally from smartphone utilization as the tethered connection enables relay over long distances and to any computer monitor.

### 3.3 Treatment Guidance

Treatment guidance imaging systems may have many overlapping requirements with diagnostic applications but primarily differ in that they have more stringent requirements for real-time visualization and analysis to provide active procedural guidance and support more rapid clinical decision making. Recent reports for SBI systems and applications that involve treatment guidance include image-guided surgery,^18^^–^^20^^,^^22^^,^^37^^,^^38^^,^^41^ management of severe burn injuries,^40^^,^^95^ PDT,^6^^–^^9^ and venipuncture.^63^^,^^136^

The starkest example of SBI for treatment guidance is surgery. Because surgical procedures are very costly in terms of medical infrastructure and human resource requirements, the typical justification of smartphone utilization based on low-cost becomes irrelevant, and usability merits of the form factor, interactive interface design, and networking capabilities of the smartphone platform must clearly take precedence. In 2014, Teichman et al.^19^ proposed the use of a smartphone app to take images and subsequent spatial measurements using software to postoperatively verify placement of toric intraocular lenses during cataract surgery. While feasibility of SBI in this application was demonstrated, no substantive assessment of clinical outcomes was undertaken and the approach does not appear to have been widely adopted. In 2018, a team of neurosurgical clinicians reported a retrospective analysis on the use of a smartphone-based rigid endoscope to visualize the surgical field in over 42 neurosurgical procedures over a span of five years. The study was a nonrandomized retrospective analysis and was limited to a qualitative case report of the usability of the smartphone in this application. The authors noted that the placement of the smartphone screen in front of the endoscope made it “more intuitive” and “enhanced 3D perception” during operation. The attachment utilized with the rigid endoscope appears to have since been discontinued.^137^ Overall, these clinical assessments of SBI systems in surgery were quite small scale and qualitative in nature, perhaps indicating a lack of confidence in providers and ethics committees to evaluate these techniques in a more substantive manner.

In two of the aforementioned applications (severe burn management and PDT), SBI systems have been proposed to provide noninvasive, quantitative quality assurance of treatment procedures which, in current clinical workflows, require less expertise to deliver but can be more subjective in nature. These applications are much better suited to leverage SBI systems. In photodynamic therapy, for example, Ruiz et al.^6^ developed a quantitative, handheld fluorescence imaging system for PpIX-PDT dosimetry before, during, and after phototherapy. The system consists of a battery powered case attachment which includes an embedded LED ring (405-nm illumination) and 600-nm longpass emission filter, and the cylindrical enclosure enables contact-based measurement at a fixed working distance and focal length, which helps ensure easy operation and reproducibility of measurements. The system was assessed using intralipid phantoms with known PpIX concentrations as well as in an animal model for PDT treatment and showed excellent quantitative precision in both applications. A clinical evaluation of another SBI system for photodynamic therapy of oral lesions at a medical center was recently reported by Khan et al.^9^ to assess low-cost technological treatments for this disease. Twenty-nine patients with confirmed oral squamous cell carcinoma lesions and who were undergoing PDT were imaged using white light, ultrasound, autofluorescence, and PpIX fluorescence imaging pre- and post-treatment. Fluorescence imaging guidance was demonstrated to provide visual guidance to demarcate lesion boundaries and quantitatively confirm the treatment region pre- and post-treatment due to photobleaching. The authors noted some practical limitations of their current instrumentation (offline analysis routines as well as the handheld device being too bulky for easy access to all regions of the oral cavity), but it is more evident that the clinical assessments were rigorously performed in the intended clinical setting and that future developments will continue to leverage SBI in appropriate and meaningful ways.

## 4 Discussion

The pervasive rationale for smartphone utilization in biomedical imaging has emphasized a multitude of factors including cost, portability, connectivity, ease-of-use, and scalability. While these factors are clearly desirable features of biomedical imaging systems, rigorous justification for how SBI systems outperform non-SBI systems is often lacking. The ubiquity of smartphones is frequently cited in literature as a justification that SBI systems are inherently low-cost, easy-to-use, and scalable biomedical imaging solutions. However, undermining this claim is the reality that the majority of original research for SBI systems is limited to a single phone model and utilizes manual, often fragmented image acquisition and analysis pipelines. While this is partly the nature of research prototyping, it is important to openly discuss these limitations and continually move toward practices that will enable greater reproducibility and translation of research advances in SBI.

### 4.1 Guidelines for Appropriate Use of Smartphone for Biomedical Optics

As discussed in the prior sections, smartphone-based hardware and software interface design varies greatly, with some designs more fully/appropriately utilizing the capabilities of the smartphone platform and others less so. Here, we provide six keys (the six C’s) as recommended guidelines to assess the appropriateness of smartphone use in biomedical imaging systems.

Clinical context represents an understanding of the clinical need and the intended user. These factors should always provide the overarching context for device design and development. Smartphone utilization should primarily be justified within this context and if the workflow needs of the clinic match the capabilities of a SBI system, or if other more dedicated systems would be superior. The ideal scenario is to assess new devices in their intended setting (at home, diagnostic lab, health clinic, central hospital, etc.) and in the hands of the intended operator (patient, lab technician, nurse, physician). When that is not feasible, device developers should be careful not to overstate the impact of their systems and acknowledge this limitation.

Completeness represents achieving a complete implementation of the intended clinical workflow, including custom app development and use and testing by nonresearch personnel. Manual image processing steps using desktop software or evaluations performed only in lab settings are often signs that a complete workflow has not been achieved. Achieving a complete implementation is clearly the best way to test the value of the approach.

Compactness relates to the importance of portability and small size for the intended application. For diagnostic and treatment guidance systems, if handheld use and/or easy transport between exam rooms will enhance usability, smartphone use is more appropriate. Alternatively, if the use case is in healthcare outside the medical center, or in remote setting, does the compact nature of the phone and the addition match the economics and portability needs of the situation?

Connectivity refers to the importance of wireless communication for the intended application. Applications that use wireless communication, through either Bluetooth or Wi-Fi, and features such as cloud computing for centralized data are ideal for SBI systems. Scalability and multiuser deployment are appropriate choices for these as well.

Cost is often the most emphasized reason for development of SBI systems, but it should be noted that this is rarely a true argument. Low-cost optoelectronic systems are now widespread and the cost of the most advanced smartphones has grown. The cost issue should be carefully evaluated, particularly if the application requires a hardware attachment and is intended for diagnostic use or treatment guidance. In these contexts, costs associated with regulatory approval and marketing will far outweigh material costs. Designs that prioritize the aforementioned C’s as opposed to minimizing prototyping costs are more likely to make an impact in medicine.

Claims refers to general statements regarding ease-of-use, cost, or scalability of SBI systems by virtue of smartphone utilization alone. Such claims should not be promulgated in research literature but rather appropriately justified through quantitative assessments. Some studies have quantified usability improvements by measuring time for task completion, performing surveys, or conducting blinded image reviews.^11^^,^^129^^,^^133^ Broader use of such assessments to substantiate improved usability of SBI systems should be encouraged. When possible, usability should be assessed in the intended clinical setting, but anatomical models and/or imaging phantoms can be good alternatives when clinical evaluation is infeasible.^138^

**Fig. 5:**
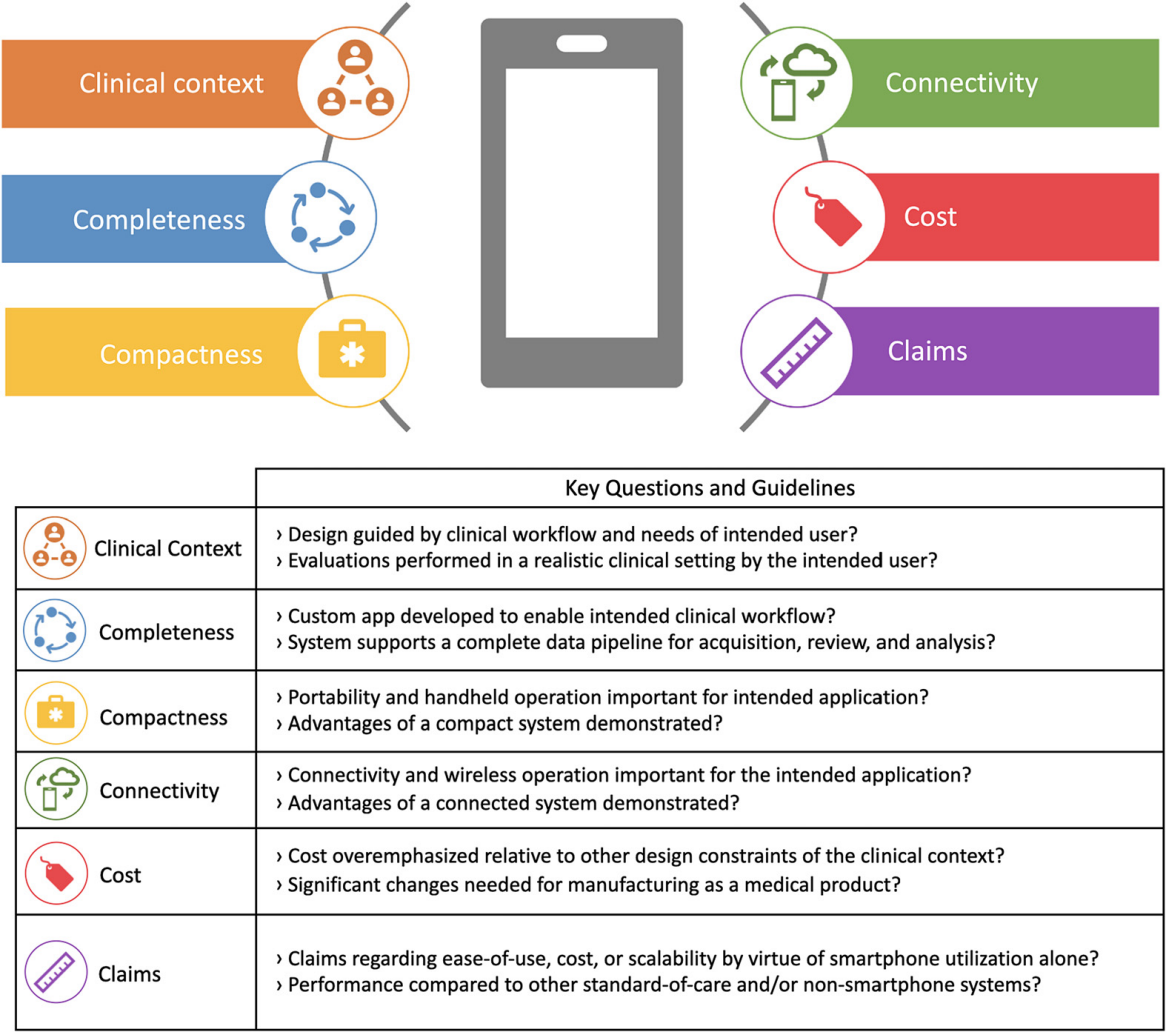
Six guidelines for evaluating appropriateness of smartphone utilization in biomedical imaging applications.

Key questions to embody the six proposed guidelines are contained in Fig. 5. These questions are intended to be used as a self-assessment for biomedical optics developers to encourage more careful consideration of the design choices made during SBI system development and improve the overall quality and rigor of SBI system assessments reported in the literature.

Ultimately, smartphone utilization in biomedical imaging is a multifaceted design choice, which should be carefully justified and evaluated by system developers. That is to say, any biomedical imaging system can, in theory, be prototyped with better overall optical and computational performance using scientific-grade components. On the other hand, many systems prototyped as “low-cost” systems on smartphones can likely be implemented at even lower fabrication cost with greater reproducibility using single board computers or embedded processors connected to peripheral camera sensors.^80^^,^^139^^–^^143^ Therefore, the burden should be placed on SBI system developers to demonstrate the unique advantages of their systems through prospective clinical assessments. Achieving this will require working toward greater reproducibility and translation of SBI systems.

### 4.2 Achieving Reproducibility and Translation of Smartphone-Based Optical System Design

In the past decade, several startups have launched SBI products targeted toward dermatology and ophthalmology clinics as well as telehealth applications, but none have yet gained significant traction in medical practice. With over a decade since SBI systems for healthcare applications have been under development, the lack of commercial success for SBI systems should raise concern. While it is challenging to comprehensively identify barriers to translation of SBI systems, we postulate that the speed at which smartphone technology evolves and the short lifecycle of these products is not readily conducive to medical device manufacturing standards. For hardware interfaces, new form factors and camera modules are launched each year, necessitating redesign of optical attachments. The lack of standardization in software development and reproducibility of results for SBI are a barrier for research progress. Here, we propose the following three items to move toward wide adoption of SBI systems: (1) focusing on hardware design that facilitates adoption of varying phone models, (2) creation of open-source software for SBI system development, and (3) adoption of robust calibration methods to best facilitate quantitative reproducibility.

Hardware design that focuses on attachments that are adaptable to different placements of the camera will be imperative for this field to gain long-term traction. Alternatively, the cost of attachment development could be sufficiently low as to allow ease of development for multiple platforms, similar to the smartphone case marketplace today. Today most devices are made for a specific phone model and customized around it, but further thought into adaptive design for constant changes in camera placement and phone sizes will be important. Hardware is more difficult to standardize as people will likely elect to use different smartphones for development. As a starting point, sharing of CAD files for optical attachments, custom enclosures, and electronic schematics with publication should be encouraged. Many research groups and startups focus on 3D printing of the hardware containers which is now a reliable and reasonable way to prototype. The shift from 3D printing technology to automated production of attachment hardware via machining, injection molding, or thermosetting will likely be important. The attachments with optical components can take advantage of highly developed optomechanical engineering that has already revolutionized the smartphone camera industry. The major benefits of spectral, polarization, or gated sensing and imaging remain to be fully exploited with custom attachments.

Open-source software toolkits and starter applications for biomedical imaging are a good place to start addressing existing development and reproducibility problems in SBI. Effective smartphone app development and maintenance requires significant programming expertise and is currently a barrier for many researchers who might be developing their software from scratch. In order for research prototypes to achieve clinical translation, standardized methods for SBI software development are needed. Cho et al.^144^ proposed a concept for a “retargetable application development platform for healthcare mobile applications.” Such a project is a worthy goal. In another recent review on smartphone point-of-care adapters, Alawsi and Al‐Bawi proposed that cross platform app development using Ionic or Xamarin as a possible solution.^53^ Although cross-platform app development could help in principle, it would likely be limited to only the subset of functionality which is common to all operating systems and would utilize the phone’s built in compression algorithms. This would not be ideal for quantitative fluorescence imaging for example. An alternative starting point is to create and maintain platform-specific templates that support core functionality needed for biomedical imaging which would include support for RAW image acquisition and standardized processing routines for common biomedical image analysis tasks.

Robust calibration of SBI systems is essential for addressing reproducibility problems and achieving clinical translation. Two major factors in this regard are: (1) lack of characterization of sensor performance (dynamic range, SNR, absorption spectra of built-in filters, demosaicing, and data acquisition rates) and (2) “black-box” processing that phones perform on the CMOS imaging data to generate traditional 8-bit RGB images. The use of RAW pixel data to confirm suitable processing pipelines is a straightforward way to circumvent this issue for all applications, including colorimetric and quantitative techniques. Given the increased complexity of accessing and analyzing RAW pixel data, suitable alternatives include color and gray-scale calibration targets (X-rite ColorChecker, for example).^67^^,^^69^ Moving toward full system characterization using radiometric calibration methods to understand results presented in studies should also be encouraged. Relative radiometric calibration methods for smartphones have been proposed.^70^ Absolute radiometric calibration methods that are optimized for SBI systems should be developed to aid in the development of quantitative applications such as fluorescence imaging. Additionally, public or app-specific sharing of image measurements from commercially available optical phantoms/targets can help ensure reproducibility of optical measurements. Development of easily networked access to file spaces will enable platforms that take advantage of off-phone computing resources such as deep learning algorithms that interpret the image data. At a minimum, these steps would enable relative calibration and comparison across hardware systems in the literature.

## 5 Conclusions

SBI systems have demonstrated a large array of applications and exhibit great potential to facilitate compact, easy-to-use biomedical imaging systems. However, for SBI systems to achieve that potential, more holistic assessments of SBI systems are needed to enable greater reproducibility and demonstrate value within their intended clinical settings. Evaluation of SBI systems should take into account clinical context, completeness, compactness, connectivity, cost, and claims associated with novel systems. Claims regarding the scalability and low-cost of SBI systems based on the ubiquity of smartphones should not be sufficient to justify their novelty and impact. Ongoing work in SBI for medical applications should prioritize realistic clinical assessments with quantitative and qualitative comparisons to other non-SBI systems in order to more clearly demonstrate the value of SBI systems within their intended applications. Improved hardware design to accommodate the rapidly changing smartphone ecosystem, creation open-source software and starter applications for SBI system development, and adoption of robust calibration techniques to address phone-to-phone variability are three high priority areas to move SBI research in biomedical imaging forward.
